# Preclinical validation of therapeutic targets predicted by tensor factorization on heterogeneous graphs

**DOI:** 10.1038/s41598-020-74922-z

**Published:** 2020-10-26

**Authors:** Saee Paliwal, Alex de Giorgio, Daniel Neil, Jean-Baptiste Michel, Alix MB Lacoste

**Affiliations:** 1 BenevolentAI, 1 Dock72 Way, 7th Floor, Brooklyn, NY 11205 USA; 2 BenevolentAI, 4-6 Maple Street, Bloomsbury, London, W1T5HD UK

**Keywords:** Target identification, Data integration, Machine learning

## Abstract

Incorrect drug target identification is a major obstacle in drug discovery. Only 15% of drugs advance from Phase II to approval, with ineffective targets accounting for over 50% of these failures^1–3^. Advances in data fusion and computational modeling have independently progressed towards addressing this issue. Here, we capitalize on both these approaches with Rosalind, a comprehensive gene prioritization method that combines heterogeneous knowledge graph construction with relational inference via tensor factorization to accurately predict disease-gene links. Rosalind demonstrates an increase in performance of 18%-50% over five comparable state-of-the-art algorithms. On historical data, Rosalind prospectively identifies 1 in 4 therapeutic relationships eventually proven true. Beyond efficacy, Rosalind is able to accurately predict clinical trial successes (75% recall at rank 200) and distinguish likely failures (74% recall at rank 200). Lastly, Rosalind predictions were experimentally tested in a patient-derived *in-vitro* assay for Rheumatoid arthritis (RA), which yielded 5 promising genes, one of which is unexplored in RA.

## Introduction

The majority of Phase II and Phase III clinical trials fail due to lack of efficacy^4^. From 2013 to 2015, efficacy failures accounted for the termination of 48% of Phase II and 55% of Phase III clinical trials, with stoppages attributed largely to incorrect drug target identification^2^. Over the past 200 years, only about 1,500 drugs cleared clinical trial and reached approval, leaving the majority of nearly 9,000 diseases without the possibility of treatment options^5^. These failure rates incur a huge financial and societal cost, and highlight a need for improved approaches to selecting more effective drug targets at the early development stage, a process known as *gene prioritization*^6^. Gene prioritization algorithms aim to extract signals of disease relevance from varied data sources, so that fewer, higher quality targets can be tested^7–9^. By using large-scale data fusion and inference, these methods harness the power of an exponentially growing body of scientific literature, in addition to integrating a wide range of experimental data sets^10,11^.

Most current methods for gene prioritization are based on “guilt-by-association,” or the principle that similarity to known drug targets of a disease can help identify valuable new gene-disease relationships. While guilt-by-association can produce reliable predictions^8^, similarity-based algorithms typically struggle to make predictions for diseases with few known associated genes^10^, and have difficulty combining and resolving conflicting evidence. As more advanced computational methods with the ability to model complex networks develop, the value of integrating heterogeneous data sources increases. The most promising computational approach to gene prioritization has been matrix factorization, in which entities (e.g. diseases, genes) and their association types (e.g. therapeutic relationships) are represented as an incomplete matrix, with the goal of filling in missing links, a process known as relational inference^10, 12, 13^. Tensor factorization, an enhancement of matrix factorization, provides two main advantages. First, the explicit representation of multiple entity relations, afforded by the 3-dimensional nature of tensor factorization, enables easy aggregation across heterogeneous data sets. For example, each data source can be modelled as a different relationship type between entities, allowing for the integration of confirmatory and conflicting evidence and thereby decreasing the false positive rate. Second, tensor factorization extends matrix factorization by allowing latent representations of these entity relations, resulting in greater generalizability, particularly for diseases with little data.

In this paper, we introduce a novel method for gene prioritization, Rosalind, that combines relational inference via tensor factorization with graph-based data integration to predict disease genes. Rosalind’s knowledge graph extracts data from heterogeneous sources, including literature evidence, differential expression, and clinical trial data, and consists of entities connected through relationships (such as ’therapeutic relationship’ or ’biological association’). In Rosalind, a tensor factorization model is trained on this heterogeneous knowledge graph to produce a ranked list of genes for every disease. Rosalind uses a state-of-the-art scoring function (ComplEx^14^) that enables the modelling of asymmetric relationships between entities. To the best of our knowledge, this is the first application of graph inference via tensor factorization with ComplEx for gene prioritization. Rosalind out-performs five comparable approaches in identifying those drug targets most likely to be therapeutically linked to a disease. Rosalind is able to make prospective predictions using time-sliced data^15^ as well as predict those genes that have a high probability of efficacy in a clinical trial^16^.

As an experimental validation, a ranked set of drug targets produced by Rosalind for Rheumatoid Arthritis (RA) was tested in a patient-derived assay. In RA, Fibroblast-like synoviocytes (FLSs) that proliferate in the joints of patients produce cytokines that recruit pro-inflammatory cells. Approximately 40% of patients do not respond to the current best treatment, anti-TNF drugs^17^; for those patients who do respond, FLSs can re-initiate disease upon cessation of anti-TNF treatment. Therefore, drugs inactivating FLSs could produce longer, more sustained responses than anti-TNF alone. 55 of the top Rosalind-scored targets for RA were tested for their ability to inactivate FLSs. Several promising targets were identified, including one drug target currently unexplored in the context of RA, MYLK, and four drug targets with few prior links to RA. Additionally, genes tested in the assay showed an efficacy comparable to that of an assay run by Jones et al. using similar conditions^18^ but testing well-established genes for FLS inactivation in RA. Taken together, these findings demonstrate the ability of Rosalind to predict therapeutic targets that show efficacy in a patient-derived *in vitro* screen.

## Results

### Performance of Rosalind

**Figure 1:**
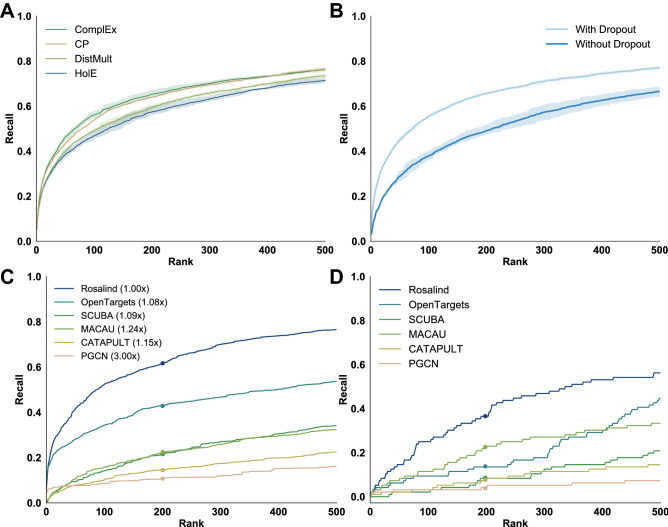
Comparison with link prediction methods. (**A**) Performance comparison of decoders. The curves plot the mean recall on a held-out test set of gene proteins across the benchmark diseases for four widely-used tensor factorization algorithms. Shaded areas indicate +/- 1 standard deviation across 3 random seeds. As rank is increased, recall of the correct predictions monotonically increases, resulting in the characteristic curves seen here. (**B**) Performance both with and without dropout’s regularizing effects on the entity embeddings, indicating mean and +/- 1 standard deviation of recall on the held-out test set gene proteins. (**C**) Performance of Rosalind against other state-of-the-art gene prioritization methods for 198 diseases. As not all algorithms support predictions for all of the test diseases, a score multiplier shown here in parentheses is applied based on the number of correctly-grounded diseases to account for the missing recall values of unsupported diseases (e.g. if only half of all diseases could be mapped, a 2x multiplier was applied to recall). (**D**) Performance of Rosalind against other gene prioritization algorithms for the specific disease of Rheumatoid Arthritis. The discretized nature of the plot is due to recalling individual gene proteins at particular positions; unlike previous plots, this is not averaged across multiple diseases.

Rosalind was trained on a biomedical knowledge graph consisting of 5 entities (Disease, GeneProtein, Compound, Mechanism and Pathway) connected by biologically meaningful relations indicating biological associations, literature evidence, and therapeutic relationships. In order to condition the model to learn to predict those drug targets most likely to be therapeutically linked to a disease, Rosalind used a subgraph consisting of Disease-GeneProtein links with relation ‘Therapeutic Relationship’ as a benchmark. The model was trained on the full knowledge graph, and was evaluated (via a validation and test set) on this Therapeutic Relationship benchmark. Disease-Disease and Compound-Compound relationships were not included, as the former were not available across enough of our diseases of interest, while the latter were not available for measures of functional similarity at sufficient resolution.

The state-of-the-art comparison was done in two stages. For the first stage, the performance of the scoring function used by Rosalind, ComplEx, was compared with three comparable scoring functions used in matrix factorization methods: DistMult, canonical polyadic factorization (CP), and holographic embeddings (HolE). These functions, or decoders, combine latent embedding representations of entities and relations into a score that relates to the probability of that edge existing. The output of the decoder is then used to determine the rank assigned to a gene for a particular disease.

In the second stage, Rosalind as a whole (data and model) was compared to similar gene prioritization methods. For this comparison, the benchmark dataset was refined using the procedure outlined in Zakeri et al. 2018^10^ by first limiting our diseases to the 314 used by Zakeri et al., 2018, and then further constraining to only diseases with a GeneProtein-Disease node degree in our Biomedical Literature Database of 30 or greater, resulting in 198 final diseases and a total of 4,613 GeneProtein-Disease Therapeutic Relationship edges. In order to evaluate Rosalind’s performance against comparable algorithms, we compared performance on this 198-disease test set.

In the first comparison to the state of the art, the performance of Rosalind’s ComplEx decoder was measured against three alternative decoders: CP, DistMult, and HolE (see Methods for description). The choice of the ComplEx decoder contributed slightly to performance (ComplEx recall@200 was 65.30% compared to CP at 64%, DistMult at 59.63% and HolE at 57.46%), with the largest difference in performance occurring approximately between rank 100 and 200 (Fig.  1A). Including or excluding dropout had the greatest impact on performance (65.62% recall@200 with dropout versus 49.08% recall@200 without; Fig. 1B). Performance was measured across the full test set.

In the second stage of this analysis, Rosalind performance (that of the combination of dataset and model) was compared with alternative published gene prioritization algorithms. A detailed description of the methods of comparison can be found in the Supplementary Information. Overall, Rosalind outperformed other state-of-the-art methods (Fig. 1C), with a recall@200 of 61.5%, followed by OpenTargets (42.96% recall@200), SCUBA (21.66% recall@200), MACAU (21.87% recall@200), CATAPULT (14.56% recall@200) and PGCN (10.55% recall@200). Evaluation using a different metric, mean average precision at rank 500 (mAP@500) also suggests Rosalind outperforms these five existing methods (Supplementary Table 5). Due to disease name grounding and data alignment issues, recall across algorithms was scaled with a multiplier, shown in the legend of Fig. 1C. This could result in an advantage for the externally-derived models. The same performance order is observed when comparing performance on the 40 diseases we were able to ground in all models (Supplementary Figure 2, Supplementary Table 6), as well as RA alone (Fig. 1D). A full description of all algorithms and multipliers can be found in the Methods section.

### Time-sliced performance

**Figure 2:**
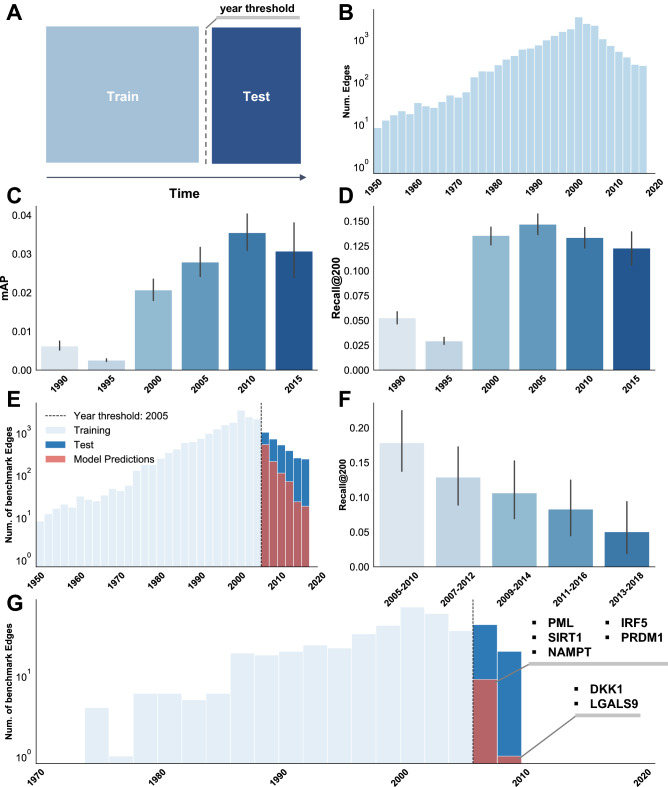
Time-slicing results. (**A**) Schematic of the split between training and test data. (**B**) Histogram of Therapeutic Relationship Benchmark edges per year in the full training and test sets. Note that each data point denotes the first literature mention of each edge. (**C**) Rosalind mAP on a time-bound benchmark. The year thresholds used to separate training data from test data are shown on the x-axis. Time-bound test sets were limited to a 5-year window after but not including the year threshold (i.e. training data for Rosalind time-sliced at 2010 contains edges up to and including 2010, and test data includes edges from 2011 to 2015 inclusive). Rosalind was trained separately on each training dataset, and evaluated on each corresponding test set. Recall@200 for these splits is shown in (**D**). (**E**) Histogram of year-tagged Therapeutic Relationship Benchmark edges with 2005 year threshold indicated. In light blue are the edges that were in the training data, in dark blue is the test set. Benchmark targets correctly predicted by the time-sliced model are shown in red. Here, the time-bound benchmark is not used, rather, all benchmark edges beyond the year threshold are used for evaluation. (**F**) Drop in recall for a sliding window of 5-years, starting at 2005. Each time window is exclusive of the first year and inclusive of the last (i.e. a 2005-2010 time window includes all dates from 2006-2010 inclusive). (**G**) Shown in light blue is the therapeutic benchmark relation training data for RA. Shown in dark blue is the therapeutic benchmark relation test set for RA. The test set here is time bound to a 5 year time band (i.e. 2006-2010 inclusive). Genes highlighted here are the correctly identified benchmark targets in the top 500 Rosalind predictions for RA.

To test Rosalind’s ability to make future predictions, each edge in the knowledge graph was assigned a year using the earliest publication year of relevant papers in our Biomedical Literature Database. Using this year tag, the knowledge graph was "time-sliced," meaning that Rosalind was trained on data up to and including a particular year threshold and evaluated on a test set containing edges after that year. In order to compare performance of Rosalind using different year thresholds, a "time-bound" test set was created for each model, constructed using benchmark edges that fell within a 5-year window after the year threshold and used in Fig. 2 plots C and D. For performance of the full model (Fig. 2E), the full test set containing years from 2005 to 2019 was used.

To test time-sliced performance over different thresholds, six versions of the Rosalind training data were generated using year thresholds in 5 year increments between 1990 and 2015, inclusive. Rosalind performance on these time-bound test sets peaked at 3.54% mAP using a year threshold of 2010 (Fig. 2C), and recall@200 peaks at 14.7% in 2005 (Fig. 2D). Rosalind achieves 23.9% recall@200 using a year threshold of 2005 on a temporally unbounded test set (i.e. all benchmark edges with years greater than 2005), implying that Rosalind successfully identifies approximately 1 out of every 4 correct therapeutic edges in the top 200 predictions (Fig. 2E). Rosalind demonstrated an expected decay in predictive power on test set edges for each subsequent year beyond the year threshold (Fig. 2E). Recall@200 on a sliding, 5-year window starting from 2005 and progressing forward in 2-year increments drops 50% after the first five years post-year threshold, showing that Rosalind is more successful at predicting imminent discoveries than those in the distant future, as expected from a model trained on the current scientific literature (Fig. 2F).

Using RA as a case study, Rosalind’s future predictions (full model with year threshold 2005, 2006-2010 time-banded test) are inspected (Fig. 2G). Rosalind correctly identifies 8 of the 73 time-banded benchmark targets (between 2005 and 2010) within the top 500 predictions (12% recall at 500). Here, we use recall at 500 as opposed to 200 to allow us to explore the quality of our forward predictions despite the small number of year-labelled edges we have in the test set. These targets, in order of rank, are NFE2L2, PML, DKK1, SIRT1, NAMPT, IRF5, LGALS9, and PRDM1. 7 of these 8 targets have no literature evidence of a relationship with RA prior to 2005, supporting the quality of the time tagging. Inspecting these targets further: since 2005, DKK1 has been shown to modulate bone fragility in RA patients^19^, PML has been shown to inhibit Fas-mediated apoptosis of FLSs from RA patients^20^, and SIRT1 over-expression promotes pro-inflammatory cytokine production in FLSs^21^. Finally, a genome-wide association (GWAS) study conducted by Rayachaudhuri et al.^22^ demonstrated a relationship between PRDM1 and RA, and Seki et al.^23^ demonstrated the role of LGALS9 in inducing apoptosis in patient RA FLSs. The quality of these targets demonstrates Rosalind’s ability to predict promising future targets using only past data.

### Clinical trial success and failure

**Figure 3:**
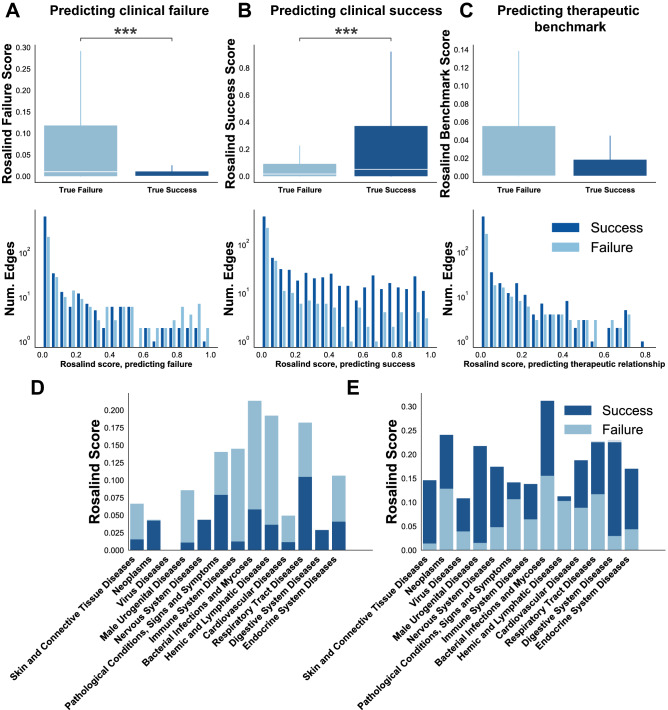
Clinical trial success and failure prediction. (**A**) Distribution of Rosalind scores for true failure versus true success when predicting on the relation Clinical Trial Failure, (**B**) when predicting on the relation Clinical Trial Success, and (**C**) when predicting on the benchmark relation, Therapeutic Relationship. For boxplots, white horizontal lines indicate the median of the distributions. The box extends between the first and third quartile (and show the interquartile range). Whiskers extend to the ends of the distribution. Plots do not show outliers. Shown below A, B and C are the histograms of the scores for clinical success and failure when predicting on the relation shown in the boxplot above. Significance was tested using an MWW test. P-values are Bonferroni-corrected for multiple comparisons, resulting in a significance threshold of $\alpha$=0.017. ***$p<$0.001. (**D**) Clinical success and failure scores across the MeSH disease tree when predicting on clinical failure. (**E**) Clinical success and failure scores across the MeSH disease tree when predicting on clinical success.

In order to demonstrate the impact of Rosalind at a later stage of the drug discovery process, Rosalind was trained on Clinical Trial Successes and Clinical Trial Failure edges extracted from Shih et al.^24^ (more detail in Supplementary Information). After training, Rosalind produced predictions on three relations: Clinical Trial Success, Clinical Trial Failure, and Therapeutic Relationship (the benchmark relation). For Clinical Trial Failure predictions, the test set contained 265 edges, with 155 unique diseases, and Rosalind achieved recall@200 of 75% across these 155 diseases, with a a mAP of 9.7%. For Clinical Trial Success predictions, the test set contained 542 edges with 338 diseases, and Rosalind achieved a recall@200 of 75% across these 338 diseases, with a mAP of 22.5%.

When predicting on the relation Clinical Trial Failure, a two-sided MWW test revealed that the Rosalind score distribution across true failures (median=0.01) was significantly greater than the score distribution for true successes (median=0) (U=89474.5, $p<$0.001) (Fig. 3A). When predicting on Clinical Trial Success, a two-sided MWW test showed that the Rosalind score distribution of true successes (median=0.05) was significantly greater than the Rosalind score distribution of true failures (median=0.02) (U=113294.5, $p<$0.001) (Fig. 3B). No significant difference was found between the scores of true successes and true failures when predicting on the relation Therapeutic Relationship (U=123170.0, *p*=0.03) (Fig. 3C). MWW tests were Bonferroni corrected for multiple comparisons, resulting in a significance threshold of $\alpha$=0.017. In order to test whether one disease area was the main driver of these statistical differences, diseases were grouped using the Medical Subject Headings (MeSH) disease tree and the average score of true success and failure was examined for each group. Figure 3D and 3E reveal that no single type of disease dominates the score distribution differences shown in plots A and B of Fig. 3.

As a case study, the top 5 clinical success predictions for RA from Rosalind (filtering out train, validation, and test data), TNFAIP3, PDE4A, EDN1, FCGR2A and GMEB1, were examined. Note that train/validation/test data for the relation Clinical Trial Success only includes edges up to April 2016, due our use of the dataset from Shih et al.^24^. TNFAIP3 was shown in 2019 to be therapeutically linked to arthritis^25^. Similarly, a PDE4A inhibitor, apremilast, was already on the market for psoriatic arthritis in 2014 and is currently being explored as a treatment for RA, with initial success in a mouse model^26^. EDN1 is highly expressed in RA patients and is linked to the excess cardiovascular mortality seen in those patients through its ability to modulate hypertension^27^. FCGR2A shows clinical relevance to RA based on its inhibition modulating treatment response to anti-TNf$\alpha$ drugs^28^. Finally, there was no literature evidence of a link between GMEB1 and RA. Overall, 4 of the 5 top predicted targets have strong mechanistic links to RA.

### Experimental validation of target predictions for Rheumatoid Arthritis

**Figure 4:**
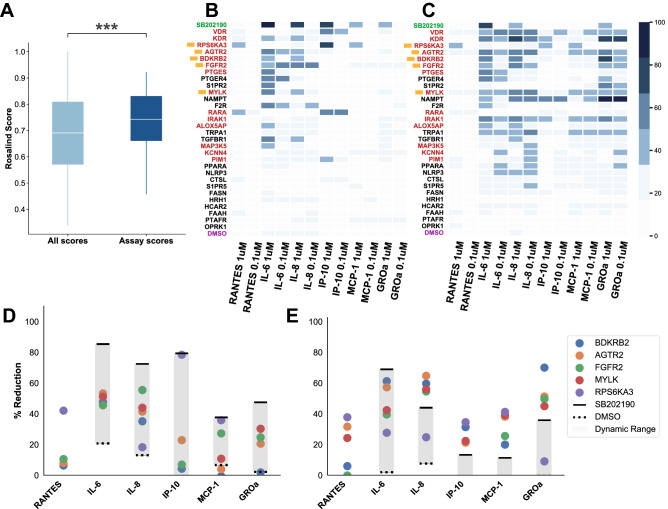
Efficacy of top-scoring drug targets in inhibiting cytokine induction in patient-derived FLSs. (**A**) Distribution of Rosalind scores for targets of compounds tested in FLSs (dark blue), and of all targets (light blue). White horizontal lines indicate the median of the distributions. The box extends between the first and third quartile (the interquartile range). Whiskers extend to the ends of the distribution, excluding outliers. Significance was tested using a MWW test; ***$p<$0.001. (**B**) and (**C**) Heatmap of the percent inhibition by immunotoxic compounds for predicted targets, showing degree of inhibition of 6 cytokines at two concentrations (1$\mu$M and 0.1$\mu$M), in cells stimulated with TNF$\alpha$ (**B**) and Poly(I:C) (**C**). In green is the positive control, SB202190, and in purple is the vehicle control, DMSO. In red are the top 14 initial hits, and the yellow squares indicate the 5 final hits of these 14 initial hits that show efficacy at 1 and 0.1$\mu$M. (**D**) and (**E**) Percent reduction in 6 cytokines of interest of the 5 final hits (colored circles) for TNF$\alpha$ and Poly(I:C) stimulation respectively at a compound concentration of 1$\mu$M. Percent reduction induced by the positive control, SB202190, is indicated by the solid black lines. The vehicle control, DMSO, is indicated by dashed black lines. Shaded gray areas indicate the dynamic range, or assay window. Unexpectedly, there was no effect of the positive control on RANTES; for this reason, our assay window excludes RANTES in (**C**) and (**D**).

To experimentally validate Rosalind’s predictions, GeneProtein-Disease hypotheses were tested in a patient-derived assay for RA. In this assay, stimulated FLSs from one RA patient were tested for secretion levels of six key cytokines implicated in the disease, identified by Jones et al.^18^, after stimulation with TNFα or Poly(I:C) and treatment with compounds associated with Rosalind predicted targets. The top 600 Rosalind target predictions for RA were selected and filtered using the triage procedure described in the Methods. Briefly, the 600 target cutoff was chosen based on observing a depletion of biologically plausible targets beyond this point. This list was reduced to the final set using characteristics linked to target progressability, including druggability and safety risk, in order to select targets with the highest chance of clinical trial success. A set of 55 compounds associated with the resulting list of 55 targets was sent to assay and tested at three concentrations, 10$\mu$M, 1$\mu$M and 0.1$\mu$M. A post-hoc MWW test of Rosalind scores for those targets prioritized for assay (median=0.74) relative to the score distribution of the original 600 targets (median=0.69) revealed that scores of genes prioritized for testing were significantly higher compared to the top 600 Rosalind scores (U=24965, $p<$0.001) (Fig. 4A), demonstrating that the higher scoring Rosalind predictions were selected for assay.

From the assay results for these 55 compounds, 25 showed signs of toxicity at 10$\mu$M and were filtered out, leaving 30 remaining (shown in heatmaps in Fig. 4B and C, along with SB202190 and DMSO). Of the remaining 30 compounds, initial "hits" were identified as those drug targets that produced a >50% inhibition of at least two cytokine endpoints by corresponding compounds at any concentration (10$\mu$M, 1$\mu$M or 0.1$\mu$M) under either TNF$\alpha$ or Poly(I:C) stimulation. Final “hits" were identified as drug targets that produced a >50% inhibition under both TNF$\alpha$ and Poly(I:C) of at least two cytokine endpoints by any corresponding compound at concentrations 1$\mu$M and 0.1$\mu$M. 14 of the 30 targets were identified as initial hits (shown in red in Figs. 4B and C). The following 5 of these 14 initial hits were identified as final “hits": MYLK, BDKRB2, AGTR2, FGFR2 and RPS6KA3. Of these five final “hits", MYLK was judged to be significantly unexplored, based on there being no available published data showing it to impact phenotypes in RA in *in vitro* assays. Shown in 4D and E are the percent reduction observed by these 5 final “hit" at a compound concentration of 1$\mu$M, compared to the positive control (SB202190) and vehicle control (DMSO).

The experiment presented here was designed based on the assay presented in Jones et al.; assay conditions were modeled on those of Jones et al., with stimuli re-optimized to the effective concentration with 90% maximal response (EC90) in our system^18^. While there are caveats to a direct, one-to-one comparison of assay results from different laboratories, efficacy information across the GeneProtein-Compound pairs with high drug target selectivity from Jones et al., (JNKi-JNK-IN-8, p38i-PH797804, IKKi-IKK16, JAKi-tofacitinib; data obtained from the Supplementary Information provided by Jones et al.) was tested for statistical differences with the efficacy across Rosalind’s final hits, across all 6 cytokine endpoints (shown in Supplementary Figure 1 plots B and D). Importantly, no significant differences were found in the distribution of efficacy of Rosalind hits (median=21.65) versus those of Jones et al., (median=28.49) under TNF$\alpha$ stimulation (MWW U=295.0, *p*=0.131) or Rosalind efficacy (median=37.90) versus Jones et al., efficacy (median=33.40) under Poly(I:C) stimulation (MWW U=356.0, *p*=0.475). MWW tests are Bonferroni corrected for multiple comparisons, resulting in a significance threshold of $\alpha$=0.025. Our efficacy in identifying unexplored targets is similar to the efficacy reported by Jones et al. in their screen for explored targets in RA. This is remarkable given the traits of drug and apoptosis resistance displayed by FLS cells^29,30^. This drug resistance is thought to come from epigenetic imprinting and the over-expression of P-glycoprotein, underscoring the multi-drug resistance phenotype characteristic of targeting this cell type^30,31^. Additionally, in the context of drug discovery, a 9% success rate (5 promising targets of 55 screened) is particularly encouraging, when compared to drug repositioning studies for RA (e.g. Hu et al., who report a 1% success rate, identifying 9 promising compounds of 888 screened^32^) or target identification in RA using experimental data (e.g. Zhu et al., who cite a 1% success rate, identifying 3 promising targets of 313 analyzed^33^).

Several lines of evidence support the potential value of these 5 "hit" genes for therapeutic development in RA. MYLK plays proinflammatory roles in other contexts^34^, including fibroblasts^35^. A non-peptide BDKRB2 inhibitor, fasitibant, was shown to be effective in decreasing the effect of bradykinins in human FLSs, suggesting a prominent role for BDKRB2 in FLS inflammatory responses^36^. AGTR2 inhibitors have been shown to ameliorate disease in a rodent model of arthritis as well as in *ex vivo* FLS cultures^37^. RPS6KA3 has also been shown to control hyperplasia of FLSs, and to impact the course of inflammatory arthritis in mice^38^. Each of these targets had been functionally validated in RA models in fewer than 3 papers and/or later than 2016, supporting the ability of Rosalind to identify under-explored yet promising opportunities.

## Discussion

Using disease-relevant biological assays is imperative if results are to translate to the clinic^39^; however, such assays can be costly and technically challenging to use for high-throughput screening. Machine learning and, more broadly, computational approaches to gene prioritization and target identification, can allow for more directed screens of likely candidates by learning from large quantities of data. While the combination of aggregated, heterogeneous data and tensor factorization allows Rosalind to be a flexible, powerful inference engine, there are also downsides to knowledge-graph-based computational approaches to gene prioritization. One common issue with biological knowledge graphs is the presence of noisy (i.e. erroneous) data, limiting the power of relational inference algorithms. While Rosalind uses established biomedical databases and expert-validated text-extracted data for knowledge graph construction, these sources are noisy: biological data is often ambiguous or even contradictory. Time-tagging for the retrospective results and assignment of phase II success or failure are also imperfect sources. In fact, inspecting the targets for RA, we find one incorrect year tag for a target in the benchmark. Additionally, all unknown Disease-GeneProtein links are considered "negative", however, true negative data (i.e. edges that are plausible but false) could improve the ability of Rosalind to distinguish therapeutic targets. Unfortunately, such data in biology is difficult to come by, and since the absence of results does not necessarily indicate negative results due to experimental factors, experts are typically reluctant to identify true negatives. More advanced methods for negative sampling (e.g. adversarial negative sampling, as in Wang et al., 2018^40^) and the explicit modeling of uncertainty could help address these issues. Future work could improve negative sampling for Rosalind, and explore methods of evaluation designed for positive-unlabelled data sets^41.^

A second common challenge with knowledge-graph-based methods is the lack of interpretability of predictions, that is, the ability for the algorithm to provide a rationale for its scores. Recent studies using graph convolutional neural networks for link predictions address both the noise and interpretability challenges^42,43^, however, more work is needed to make them practically useful in gene prioritization. A third challenge with approaches like Rosalind is the potential for "easy inference", i.e. a model prediction based on either simple pattern-matching or true data leakage between training and test sets, instead of a meaningful representation of the problem. It can also lead to an overly-optimistic assessment of performance. By restricting the set of diseases to those that have more than 30 genes in the benchmark in order to avoid evaluating on disease subtypes, we partly mitigate this problem. A next step to addressing this issue would be to model disease hierarchies, and create train-test splits based on a particular level of disease granularity.

Finally, a third common pitfall of knowledge graphs methods is that they often lack granularity in their entity relationships, due to the coarseness of the databases from which they derive, which can lead to predictions with low specificity. For example, the KEGG database provides only one kind of gene-disease association, which can in fact reflect a range of underlying relationships, from a human genetic link to an association in a disease model. In addition, protein-protein interaction databases often do not consider different tissue types. This can be a problem for diseases in which differences in gene interaction networks in a specific organ or cell type affected is often critical to understanding the disease pathophysiology and treatment potential. In addition, many disease pathways are missing from existing databases. One can address these data issues by augmenting knowledge graphs with expert-curated data, or specific text extraction^61^. Another way to address these issues is to use post-processing steps, and leverage a variety of metadata such as tissue expression or pathway involvement to filter target predictions.

## Conclusion

Here, we introduce Rosalind, a novel, holistic approach to gene prioritization that combines data integration with tensor factorization to predict therapeutic genes for diseases. Rosalind outperforms state-of-the-art gene prioritization algorithms in predicting those genes likely to be therapeutic targets (Fig. 1) and can make prospective predictions using time-sliced data (Fig. 2). Using tensor factorization to explicitly model both entities and relations affords Rosalind the unique ability to learn and distinguish subtle and important relationships: we show that Rosalind is able to distinguish between those genes most likely to fail and succeed in clinical trials by learning the nuances of the clinical success and clinical failure relations (Fig. 3). Finally, we demonstrate the *in silico*-to-*in vitro* translatability of Rosalind’s predictions by evaluating 55 top-scoring genes for Rheumatoid Arthritis in cell assays, allowing us to identify both known and novel potential drug targets for RA (Fig. 4) with efficacy comparable to that of those presented in Jones et al.^18^. This is particularly encouraging because the molecular inhibitors used in Jones et al. hit well-established immune targets, underlining the ability of Rosalind to identify promising targets that are little explored in the literature.

Improved gene prioritization methods such as Rosalind avoid using costly, large-throughput screens and, in their place, enable streamlined, directed assays testing evidence-based hypotheses that have a higher probability of downstream success. Overall, Rosalind provides a flexible, improvable approach to gene prioritization that is able to generate clinically relevant predictions at scale. This could allow it to generate promising clinical avenues for thousands of diseases with unmet need, and to help slow the trend of declining productivity in drug discovery.

## Methods

### Knowledge graph construction

**Figure 5:**
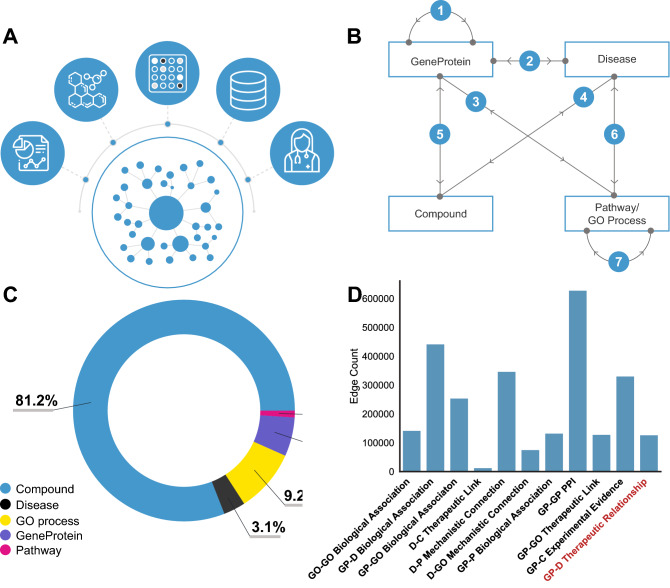
Knowledge graph construction. (**A**) A heterogeneous knowledge graph is constructed from literature evidence, compound libraries, experimental data, databases of collated and curated datasets, and clinical trial evidence (**B**) A canonical set of connections in the graph. Note that these edges can be directional: **1** GeneProtein entities are connected to other GeneProtein entities through protein-protein interactions (PPI). **2** GeneProteins are connected to Diseases through Therapeutic Relationships (benchmark) and Biological Association relations. **3** GeneProteins are connected to Pathways via Biological Association relations, and are connected to GO processes via a Biological Association link and a Therapeutic Link relation. **4** Compounds are connected to Diseases via Therapeutic Links. **5** GeneProteins are connected to compounds via Experimental Evidence edges. **6** Diseases are connected to Pathways and GO processes via a Mechanistic Connection relation. **7** GO processes are connected to GO processes via Biological Association relations. (**C**) The percentage compositions of the five biomedical entities is shown here, with Compounds dominating the graph. (**D**) The number of edges between each pair of entities. GP=GeneProtein, GO=GO process, D=Disease, C=Compound, P=Pathway. Shown in red is the benchmark relation, GeneProtein-Disease Therapeutic Relationship.

The knowledge graph component of Rosalind consists of biomedical entities and directed relations that link them together, and is constructed from both structured and unstructured data (Fig. 5A). An entity-relation-entity triple is referred to as an *edge* in the graph, and has a binary representation (1 if the edge exists, 0 if it does not). This graph is then separated into three disjoint data sets: training data, validation data and test data. Rosalind is trained on the training data set, and its performance on the validation data is used to determine when to stop training. Training is stopped when metrics calculated on the validation set do not change over 5 training epochs, implying that the model has fully extracted all useful information from the training data. Rosalind’s final performance statistics are then evaluated on the test set. Our training data span all entities and relations.

#### Biomedical literature database

Approximately 20% of our knowledge graph edges are extracted from the literature. The corpus used for text extraction is a combination of public and licensed documents. It is composed of 29 million PubMed abstracts, 1.5 million PubMed Central (PMC) open-access full-text articles for commercial use and 4.1 million licensed full-text articles from multiple publishers. The documents are merged based on PubMed/PMC/DOI information to avoid duplicate data in information extraction.

#### Named entity recognition

In order to extract relevant information related to biomedical entities in structured data, a dictionary-based Named Entity Recognition (NER) method is used to extract entity names in the Biomedical Literature Database described above. In this process, abbreviations and synonyms are detected and linked together. This dictionary-based NER pipeline allows for the quick editing of data and augmenting the literature discovery process.

#### Biomedical entities

There are 5 types of entities in the knowledge graph: GeneProteins (also referred to as genes or targets), Diseases, Gene ontology processes (GO Process), Pathways, and Compounds (Fig. 5B).

GeneProteins are a set of 19,197 unique gene names from the HUGO Gene Nomenclature Committee (HGNC), using the HGNC ID as the primary identifier. Only human protein coding genes are extracted. Three additional structured data sources are used to retrieve additional synonyms and data source IDs: Entrez Gene from the National Center for Biotechnology Information (NCBI)^44^, the Ensembl genome browser Release 97^45^, and the Universal Protein Resource (UniProt)^46^.

Diseases are extracted from several sources. The Medical Subject Headings (MeSH) ontology^47^ is the seed to which all other sources are merged. The following disease sources are aggregated using this seed: Online Mendelian Inheritance in Man database (OMIM)^48^, the Disease Ontology (DO)^49^, Orphanet^50^, the National Cancer Institute Thesaurus (NCIT)^51^, and the Unified Medical Language System (UMLS)^52^. From these, 9,972 diseases are represented in the Rosalind knowledge graph.

The GO Process dictionary was downloaded from the Gene Ontology Resource on October 17, 2018^53^ and includes 29,699 terms in the “Biological Process” category, all of which are included in our training data. The Pathway dictionary is composed of a set of 302 pathway terms from the Kyoto Encyclopedia of Genes and Genomes (KEGG)^54^, release 78.2. Additionally, 2,224 pathway terms come from the Reactome Pathway Database (Reactome)^55^, version 65.

The Compound dictionary originates from ChEMBL^56^, Clarivate Analytics’ Integrity database (Integrity)^57^ and The Comparative Toxicogenomics Database (CTD)^58^. 261,812 compounds are linked and actually used in the knowledge graph.

#### Relations

The 5 types of entities described above are connected in the knowledge graph through 11 relation types constructed from multiple biomedical databases and extracted from literature. Structured data is pooled from the following sources: ChEMBL, Integrity, the National Human Genome Research Institute and European Bioinformatics Institute Genome-Wide Association Catalog (NHGRI-EBI GWAS Catalog), downloaded on February 11, 2018^59^, Disgenet^60^, CTD, KEGG, and the OMIM database.

Relation extraction from unstructured data focuses on three categories of linkages: Encode-Attend-Tag edges (EAT), a proprietary term used to describe edges that demonstrate biological association, and Literature-based Therapeutic Evidence edges (LTE) that convey a therapeutic relationship and Syntactic Subject-Verb-Object Edges (SVO), that capture directional biological associations.

EAT edges are extracted by running an algorithm that uses distant supervision to learn syntactical association on the biomedical literature database in order to extract sentences that provide evidence for a particular Disease-GeneProtein link. In addition to identifying supporting sentences in the literature, this process assigns a confidence score between 0 and 1 to each edge that reflects the accuracy of that Disease-GeneProtein link. Here, EAT edges with a score of 0.95 or higher, or the top 15% of all EAT edges, were included, amounting to approximately 300,000 edges.

LTE edges are extracted from article titles in the biomedical literature database using a set of expert-curated rules^61^. An edge is included in this dataset if a title includes both the disease and GeneProtein names, and a pre-defined lexicon that conveys a therapeutic relationship. For example, an LTE edge between ADIPOQ and Blood Platelet Disorders was extracted from the article title: "Adiponectin inhibits hyperlipidemia-induced platelet aggregation via attenuating oxidative/nitrative stress."

SVO edges encapsulate biological, bidirectional relationships between entities that exist at the sentence level. SVO extraction is done via an unsupervised, rule-based model that unearths relationships that follow strict patterns, particularly, entities linked via a verb belonging to a predefined list. SVO edges are comprised of standard subject-verb-object relationships in addition to subject-verb-in-object (i.e. GPR-9-6 [subject] was expressed [verb] at high levels IN thymus [object]) and subject-verb-to-object (i.e. Infection [subject] of DCs with live Mtb led [verb] TO cell death [object]) relationships.

#### Edge construction

Entities and relations combine to form edges, a schematic of which is shown in Fig. 5B. A summary of edges and edge counts is shown in Fig. 5D. GeneProtein-GeneProtein interactions at the protein level, that is, protein-protein interactions (PPIs), are collated from the OmniPath database^62^, the Biological General Repository for Interaction Datasets database (Biogrid)^63^, the SIGnaling Network Open Resource (SigNOR)^64^, KEGG and Reactome, resulting in a set of 629,357 edges. GO process-GO process edges are constructed using the Gene Ontology Resource hierarchy, using the following five relationships between entities: "is a", "regulates","part of","negatively regulates" and "positively regulates," resulting in a set of 143,490 edges.

GeneProteins and GO processes are connected via two relation types: the first, GeneProtein-GO process Therapeutic Link, is constructed using the AmiGO database, for a total of 129,382 edges. The second, GeneProtein-GO process Biological Association, comes from a combination of SVO edges and annotated edges, for a total of 255,265 edges. Disease-GO process Mechanistic Connection relations are constructed using gene set enrichment analyses drawn from the eDGAR database of Disease-GeneProtein associations and CTD, totalling 76,587 edges. GeneProtein-Pathway relationships are Biological Associations extracted from KEGG and Reactome for a total of 133,872 edges. Disease-Pathway edges are inferred from gene sets extracted from KEGG and Reactome, combined with GeneProtein-Disease associations from EAT. A set of filters were applied to reduce noise: for example, a Disease was required to be associated with at least two GeneProtein entities in order for a Pathway to be considered; Pathways containing names of other diseases, and Pathways with fewer than 3 genes were excluded from the analysis. This produced a total of 348,001 edges. Disease-Compound Therapeutic Link relations are derived from the Integrity database and filtered for any relation with the clinical testing phase ‘Preclinical’ or higher, resulting in 13,919 edges. GeneProtein-Compound associations were extracted from the chemical database of the European Molecular Biology Laboratory (ChEMBL) and filtered for those edges with a pChEMBL value of 7 or higher, resulting in a set of 331,852 GeneProtein-Compound edges.

Disease-GeneProtein relation are split into two broad categories. The first relation category, Disease-GeneProtein Biological Association, represents a biological association between GeneProtein and Disease. These relations are extracted from the NHGRI-EBI GWAS Catalog (downloaded on February 11, 2018), along with ChEMBL and DisGeNET, version 4.0. Additionally, EAT edges representing a biological association are included in this set of edges, for a total of 443,330 edges. The second relation category, Disease-GeneProtein Therapeutic Relationship, represents relationships between GeneProtein and Disease where the GeneProtein is causally implicated in the pathogenesis of a Disease. This relation type is constructed from the combination of CTD, KEGG, and OMIM, in addition to LTE edges, for a total of 128,018 edges.

Conclusively assessing the relative importance of each relation type to performance, however, is difficult. When removing individual relation types, the removal of GeneProtein-Disease Biological Association edges has the largest impact on performance. A full subset analysis valuing the relation types is not computationally feasible, but emerging work could help make such evaluations possible in the future^65,66^.

#### Therapeutic relationship benchmark

In order to condition the model to learn to predict those drug targets most likely to be therapeutically linked to a disease, Rosalind uses the Disease-GeneProtein Therapeutic Relationship set of edges as a benchmark, or test set, shown in red in Fig. 5. This Therapeutic Relationship relation type may include drug targets implicated in the disease by evidence derived from pre-clinical models or clinical trials, or those targets of drugs approved in the clinic. These data are included in Rosalind using the train-valid-test data split described at the beginning of this section. This means that the relation Therapeutic Relationship was included in training and solely comprised our validation and test sets. Therapeutic Relationship edges are split between training, validation and test using a 60%-20%-20% split by edge count, or a 60%-40% by edge count for those analyses in which only a training and test set were used.

For the state-of-the-art comparison, this Therapeutic Relationship dataset is further refined by following the benchmark disease refinement procedure outlined in Zakeri et al., 2018 resulting in the 314 diseases used in Zakeri et al., 2018^10^. In order to diminish the possibility of information leakage between this test set and the training data, all diseases with a GeneProtein-Disease node degree of fewer than 30 targets were removed from the test set. These diseases are often subtypes: filtering them out prevents a situation in which, for example, ALS Type 16 is in the training data and ALS Type 17 is in the test data set, artificially inflating our performance on ALS Type 17. This leaves 198 final diseases in the test set. A train-valid-test split was used and the test set was constrained to the 198 diseases in the Therapeutic Relationship Benchmark.

For reference, a summary of datasets and edge counts and entity counts can be found in the Supplementary Information, in Tables 1, 2 and 3.

### Relational inference for link prediction

In tensor factorization, each entity in the graph is represented as a *d*-dimensional vector $e \in R^d$. Similarly, every relationship link between these entities is also modeled as a *d*-dimensional vector $l \in R^d$. For a graph with *n* entities, this forms an entity embedding matrix $E \in R^{n \cdot d}$ that is learned during training, as well as a relation embedding matrix $L \in R^{k \cdot d}$ which contains all *k* relations.

Individual facts in the knowledge graph are represented as a triple: the subject, relation, and object of that fact. For example, the entities “Rheumatoid Arthritis" and the “Interleukin 6 receptor" may exist in the knowledge graph, and the statement “the interleukin 6 receptor is a therapeutic drug target in the disease rheumatoid arthritis" can be encoded by the triple (rheumatoid arthritis, therapeutic drug target, interleukin 6 receptor). The purpose of training a model, then, is to adjust the embeddings so that a given scoring function $\phi$, which combines the entities and relations, produces high scores for true facts that are present in the data and low scores for false facts that are not true according to the knowledge graph. Note that the standard “closed-world“ assumption is followed here, in which facts missing from the knowledge graph are assumed to be false^67^. Though this is of course a false declaration, as all test links would necessarily be incorrect in this framework, this assumption provides a methodology for gathering large quantities of negative samples and is commonly used in practice.

Rosalind uses the ComplEx scoring function, which operates on complex embeddings matrix $E \in C^{n \cdot d}$ and $L \in C^{k \cdot d}$. A number of similar scoring functions exist for link prediction using tensor factorization: Canonical Polyadic decomposition^68^, RESCAL^69^, HolE^70^, DistMult^71^, and ComplEx^14^, among others. The algorithms are often referred to as decoders^72^, due to their ability to decode a learned dense vector representation into the probability of a link. Because of its scalability, robust performance across hyperparameters and datasets, and ability to model asymmetric relationships, ComplEx was chosen for this work. The ComplEx decoder takes the following form:

$$\begin{aligned} \phi (s,r,o) = Re(\sum _{j=1}^d l_{rj} e_{sj} {\bar{e}}_{oj}) \end{aligned}$$

where $\phi (s, r, o)$ represents the un-normalized likelihood of the fact triple (*s*, *r*, *o*). Separating the real and imaginary parts:

1 $$\begin{aligned} \phi (s,r,o) =&\langle \text {Re}(e_s), \text {Re}(l_r), \text {Re}(e_o) \rangle + \langle \text {Im}(e_s), \text {Re}(l_r), \text {Im}(e_o) \rangle \end{aligned}$$

2 $$\begin{aligned}&+ \langle \text {Re}(e_s), \text {Im}(l_r), \text {Im}(e_o) \rangle - \langle \text {Im}(e_s), \text {Im}(l_r), \text {Re}(e_o) \rangle \end{aligned}$$

where $\langle \cdot \rangle$ indicates the trilinear product. *Re*(*x*) and *Im*(*x*) are the real and imaginary part of *x*, respectively; ${\bar{e}}$ is the complex conjugate of *e*; and *s*, *o*, and *r* are indices for the subject, object, and relation. Notably, this factored form facilitates the implementation of Rosalind in standard machine learning frameworks such as Tensorflow^73^. With these components separated, the computation becomes:

3 $$\begin{aligned} \phi (s,r,o) =&\langle e_{sa}, l_{ra}, e_{oa} \rangle + \langle e_{sb}, l_{ra}, e_{ob} \rangle + \langle e_{sa}, l_{rb}, e_{ob} \rangle - \langle e_{sb}, l_{rb}, e_{oa} \rangle \end{aligned}$$

where *a* represents the real embedding of the entity or relation and *b* represents the imaginary embedding. With this form, standard machine learning frameworks can process these embeddings separately, as if there were six real embeddings per fact ($e_{sa}, e_{sb}, l_{ra}, l_{rb}, e_{oa}$, and $e_{ob}$) instead of three complex embeddings $e_s$, $l_r$, and $e_o$.

**Figure 6:**
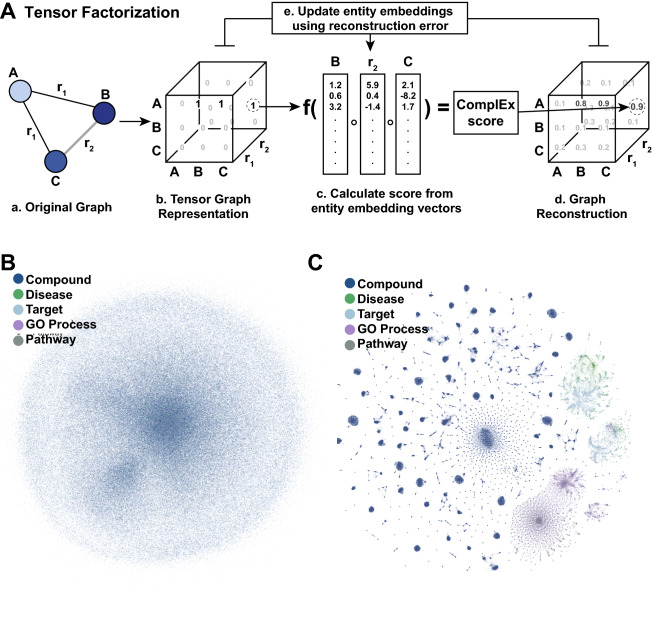
Visualization of entity representations through Rosalind. (**A**) Shown here is a depiction of training through tensor factorization, using a graph consisting of three entities (A, B, and C), and two relations, $r_1$ and $r_2$. Step (**a**) shows the original graph, with three entities connected via two relations. Step (**b**) shows the sparse tensor representation of this graph, with entities across two dimensions and relations along the third, and a 1 or 0 value for each coordinate, indicating whether an edge exists or does not exist, respectively. Step (**c**) shows the calculation of the ComplEx score for a single point in the tensor, that represents an entity-relation-entity edge, which is a function of the latent embeddings for the two entities and relation. The ComplEx score for each possible entity-relation-entity edge is calculated, and used to populate the graph reconstruction tensor (dense), shown in (**d**). Training involves calculating the reconstruction error, or the difference between the ComplEx score and the true 1 or 0 value from the original graph tensor. This error signal is then used to update the entity embeddings. (**B**) A tSNE representation of the embeddings in A before training; note the lack of structure. (**C**) A tSNE representation of the embeddings in B after training.

At the beginning of training, six embedding matrices were randomly initialized using Xavier Gaussian initialization^74^. There were $n=322,591$ entities (18,582 GeneProteins, 9,972 Diseases, 29,699 GO processes (mechanisms), 2,526 Pathways and 261,812 Compounds) and $k=11$ relations, which collectively represent 2,633,073 biological facts in the knowledge graph. The embedding dimensionality was $d=200$. For negative sampling, 5 negative facts were sampled randomly for every positive example with a matching entity type per the standard methodology. Batch sizes were 3000 examples per batch, while ensuring that there were the same number of examples between relations in every batch. This equality in number of examples per relation per batch has the effect of oversampling rarer relations, such as the therapeutic relationship specified in the benchmark relations, and consequently making those relations more important for training. Adam^75^ was used for optimizing the embeddings, and cross-entropy was used as a loss function for the optimizer. Dropout of 0.5 on the embeddings was used. Training time for Rosalind on a 4-core CPU requires approximately 33 minutes per epoch, resulting in a total of around 9 hours with 17 epochs of training and early stopping after no improvement for 3 epochs. Once trained, a batched prediction requires 74 ms per disease to predict and rank scores across all targets in total.

A visualization of the embeddings before and after training can be found in Fig. 6; note that initially random embeddings develop structure and groupings through the training process, facilitating both inference and post-hoc analysis. Note the complex, intricate structure of the t-distributed stochastic neighbor embedding (t-SNE) representations that show close embedding similarity by close distance. Certain Compounds group with other Compounds; particular Pathways group with GO processes, and certain GO processes cluster interestingly close to Diseases.

### State-of-the-art algorithm comparison

State-of-the-art comparison was done in two stages. For the first stage, the performance of the ComplEx decoder used by Rosalind was compared with three similar scoring functions used in matrix factorization: DistMult, canonical polyadic factorization (CP), and holographic embeddings (HolE). For particular datasets, CP, the oldest method^76^, has been shown to perform at the state-of-the-art with correct initialization^68^. Similarly, HolE^70^ has shown improved performance on the standard benchmark datasets WN18 and FB15k, while DistMult^71^, a recent but less state-of-the-art architecture, forms a baseline for performance. CP, HolE, and ComplEx are asymmetric decoders, meaning that the same (subject, relation, object) triple produces a different score for (object, relation, subject), while this is not true of DistMult. Additionally, for this analysis, the impact of regularization via Dropout is measured on recall performance. Dropout selects random dimensions of the embedded representation and sets the value of those dimensions to zero instead of their true values. Each dimension of the embedding has an independent probability of $p=0.5$ to be set to zero. Dropout has been shown to be an extremely effective regularizer, and decreasing overfitting helps particularly here with such highly-parameterized embedding models. For these performance assessments, data are split into train, valid, and test sets as described above, and training and validation edges ((Disease, Therapeutic Relationship, GeneProtein) triples) are filtered from the prediction list.

In the second stage, full Rosalind (data and model) was compared to similar gene prioritization algorithms. Other methods use different datasets for training and testing, and different link prediction algorithms for prioritizing which genes are the most promising for a given disease. To ensure a fair comparison, the 198-disease benchmark is used, which is a subset of the diseases used in previous studies^10^, the construction of which is detailed above. The test subset of these data is comprised of 198 diseases and a total of 4,613 GeneProtein-Disease Therapeutic Relationship edges. As alternative methods make use of different ontologies and thus represent knowledge differently, it is not possible to perfectly map the identical concepts of disease and gene directly between all methods. Similarly, it is not possible to enforce our training, validation, and testing data on external algorithms. Not enforcing a data split favors external methods, as the Disease-GeneProtein associations which comprise our test set should appear in their training data. Overall, these methods provide a qualitative comparison across five different gene prioritization methodologies, listed below:

1. Open Targets^77^, a comprehensive data-modeling-based platform that provides considerable context for each entity in order to prioritize possible therapeutic targets by integrating a variety of data sources, including genetic association data, somatic mutation data, drug information, scores derived from pathways and systems biology, RNA expression, text mining sources, and animal models.
2. SCUBA^78^, a positive-uncertain matrix completion (PU-learning) approach.
3. MACAU^10^, a Bayesian matrix factorization based approach that draws upon considerable side information, conditioning upon Interpro^79^, Gene Ontology^53^, and Uniprot^46^ for additional gene context. For diseases, literature-based disease features derived from textual term-frequency inverse-document frequency (TF-IDF) occurrences in PubMed were used, as in ElShal et al.^80^
4. CATAPULT^81^, a network-based supervised SVM-based method combined with a metric of diffusion distance on the network.
5. PGCN^13^, a graph convolutional network based approach.

As each of these alternative methods themselves show improved performance compared to a variety of other state-of-the-art methods, comparison against these methods provides a comprehensive assessment of Rosalind across gene prioritization approaches. A description of the entity alignment process across algorithms be found in the Supplementary Material. Pre-trained available models were used for Open Targets, PGCN, and CATAPULT; for MACAU and SCUBA, the models were conditioned at least partially upon Rosalind training data while reusing data from those respective algorithms. The GeneProtein-Disease links in Rosalind’s training and validation sets were filtered from the prediction lists of all methods for evaluating metrics, as per standard practice. Note the filtered GeneProteins may or may not have been actually prioritized by the models or trained upon; however, in cases where Rosalind training GeneProteins were predicted in the output of an external model, this provides a methodology to avoid penalizing models with a different training set. This establishes a favorable comparison for external models, because and external models are not penalized for training on Rosalind test GeneProteins. Finally, the performance of each algorithm is scaled according to the number of successfully mapped diseases; for algorithms such as PGCN which match only 66 of the 198 diseases, the recall score is multiplied by 3 to account for losses due to ontology mapping. The decision to choose recall as our performance metric, over alternatives such as mean average precision (mAP), area under the precision-recall curve (AUPR), area under the receiver operating characteristic curve (AUROC), accuracy, etc., was made because the data is positive-unlabelled (P-U): the positive data, or graph edges, are reasonably known, while the class assignment of “negative” data is unknown, as these edges may be discovered in the future. Recall, which is only based on pre-existing positives, is more appropriate in a P-U scenario than precision, which is dependent on accurate negative data. For this reason, recall is more representative of performance on our data, while precision, AUPR, AUROC, and accuracy would require the accurate labelling of negative data. For reference, however, mAP at rank 500 (mAP@500) values can be found in Table 5 of Supplementary Information.

### Time-slicing

To test Rosalind’s ability to make future predictions, the knowledge graph is "time-sliced", so that training and test data are temporally separated. Rosalind is then trained on data up to and including a particular year threshold and evaluated on a test set consisting of edges after that year. Edges were time tagged with the first literature mention of that relationship. For each edge in the Rosalind knowledge graph, a list of publication years of relevant articles was extracted from our Biomedical Literature Database; a document was said to contain an edge if the entities of that edge (i.e. Disease and GeneProtein) co-occur on the level of a paragraph. Each edge was then assigned the earliest year of this list, under the assumption that the first literature mention of a relationship between entities is indicative of when information of this relationship first surfaced in the scientific community. 50 edges were manually examined to evaluate whether the year tag corresponded with the earliest literature, and 74% of edges were accurately tagged. For those edges that were incorrect, there was at most a 1-year difference between the correct date and the tagged year. Note that in the non-time-sliced Rosalind model, there is no implicit temporal separation between training and test data.

Because of data quality concerns with year-tags for Compound-Disease edges and a lack of year data for GO processes and Pathway edges, only the following three relations were included in this analysis: Therapeutic Relationship Benchmark, GeneProtein-GeneProtein PPI and GeneProtein-Disease Biological Association. Overall, only 51% of these edges were tagged, resulting in a total data set of approximately 800,000 edges. Six year thresholds were selected, 1990 to 2015 in 5 year increments (inclusive), used to separate training and validation/test data (see Supplementary Table 4). For the time-banded time-slicing analysis (Fig.  2C and D) only a train and test set were used, and mAP and Recall were calculated on the full set of diseases in the forward-looking 5-year time band. While data may have been imbalanced over diseases in the various time-bound data sets due to the temporal distribution of data over time, we do not adjust for this imbalance, as temporal information leakage would be unavoidable. For example, year thresholds might have been chosen by disease to ensure an even train-test split, but because Rosalind uses information over all diseases to make a prediction on any given disease, making the model susceptible to future information leakage across diseases. Similarly, down-sampling data based on the year threshold introduces information about the future distribution of information to the training data, again causing information leakage. As this analysis is intended to simulate the performance of the system agnostic to year threshold, the data distribution was not changed. Dataset statistics on the different time bands can be found in Supplementary Table 4. For Figs. 2B, and 2E-F, the test set was constrained to the 198 diseases used in the state-of-the-art comparison; only 184 were able to be year-tagged.

### Clinical trial outcome prediction

Clinical trial outcome prediction analyses were performed using GeneProtein-Disease edges extracted from Shih et al.^24^ Supplementary Table S1 to explore Rosalind’s ability to differentiate clinical trial success from failure. Edges were constructed from data up to and including April 2016. Each edge was tagged with its maximum clinical phase and included multiple entries for each GeneProtein-Disease pair in cases where results differed based on the compound tested. For example, the target somatostatin 2 receptor is tested for acromegaly using compounds g-02113, l-363377 and ptr-3172, which achieved phases Discovery, Discontinued, and Phase II Clinical trial respectively. For these instances, the GeneProtein-Disease pair was assigned the highest phase obtained from any experiment, which, in this case, was Phase II. For the purpose of this experiment, all Phase II and Phase III discontinuations were marked as clinical trial failures and all GeneProtein-Disease pairs that achieved phase "Pre-registration or higher" as clinical trial successes. Other phases, such as Discontinued, Discovery, and Phase I clinical trial, were not assigned to success or failure since their status is ambiguous (e.g. a drug program with highest phase Discovery could be a terminated due to factors other than efficacy, such as financial reasons). As mentioned earlier, the large majority of Phase II and Phase III discontinuations are due to failures of efficacy^39^. Therefore, using only Phase II/III discontinuations data for the clinical failure category ensures that our experiment addresses efficacy as opposed to other factors affecting drug discovery success.

From Shih et al.^24^, 2,710 GeneProtein-Disease Clinical Trial Success edges and 1,327 GeneProtein-Disease Clinical Trial Failure edges were ingested, out of a possible 3,242 successes and 1,596 failures. Successes far outnumber failures due to our limiting the definition of failure to Phase II/III discontinuations. For the clinical success prediction analysis, the relations “Clinical Trial Success“ and “Clinical Trial Failure”, from Shih et al.^24^ are used as the benchmark, included in the training data and solely comprising the validation and test datasets. While it may seem that these data consist of two labelled classes, that of success and that of failure, we choose to treat success prediction and failure prediction as two separate problems. We do this because the same GeneProtein-Disease association can be represented in both clinical trial success and failure, and because a low score for success should be interpreted as less promising to succeed, but does not necessarily mean a GeneProtein-Disease pair is more likely to fail.

For all analyses, a full summary of datasets, train-val-test splits, and data statistics can be found in Supplementary Table 4.

### Rheumatoid arthritis assay

#### Target triage

The top 600 ranked Rosalind predictions for RA were assessed for inclusion. This cut-off of 600 was chosen based on observing a sequence of greater than 10 genes with no literature evidence beyond this point, suggesting limited evidence to support prediction quality. This list was refined by expert curation based on expected biological rationale, safety, tissue specificity and previous evidence. These characteristics can influence whether an eventual clinical trial is successful. Expected biological efficacy was determined using a combination of existing supporting evidence from our Biomedical Literature Database, supporting evidence from the GWAS catalog and differential expression data from the OmicSoft DiseaseLand release TCGA-B37_20190215_v8^59,82^. Safety was determined by association with adverse events in publicly available datasets, particularly, those targets with a clinical target development level^83^ were considered safer, and those involved in the absorption, distribution, metabolism or excretion (ADME) of drugs were considered unsafe^84^. Tissue specificity of a gene was assessed using $\tau$ values^85^ calculated using data from the OmicSoft database. Finally, genes were filtered for ligandability, requiring compounds to display submicromolar affinity (<1$\mu$M) for their targets from Chembl^86^, as well as being commercially available from suppliers. This resulted in a set of 55 targets and their associated compounds prioritized for testing in the assay.

#### Assay

Rosalind-predicted genes were tested in an *in-vitro* patient-derived FLS assay (BioIVT, Royston, United Kingdom). Cells were obtained from existing inventory stocks of primary human FLS from rheumatoid arthritis (RA) patients at BioIVT. BioIVT confirmed that all tissues were procured with informed consent and appropriate ethical approval for research use. Additionally, BioIVT confirms that all experiments were performed in adherence with good scientific practices according to Standard Operating Procedures. For this assay, FLSs were cultured in FGM-2 medium at passage 4. Cells were seeded in 96-well plates at 10,000 cells/well, and incubated overnight at 37$^{\circ }$C in a 5% CO$_2$ incubator. Cells were treated (n=2 experiments for each treatment) with our selected 55 test compounds. Compounds were tested at 3 concentrations (10$\mu$M, 1$\mu$M, 0.1 $\mu$M), aiming to identify dose-dependent effects, and to engage the primary pharmacology against compounds’ intended genes. This range was chosen to account for potential discrepancies between published cell-free IC50s of our compounds, and their actual potencies in the cell system. The p38 inhibitor SB202190, tested at 1$\mu$M, was used as a positive control. DMSO was used as a vehicle control, and medium alone was the untreated control. The difference between SB202190 and DMSO provided the signal-to-background ratio of our experiment. SB202190 was chosen as a positive control, as it has been shown to significantly control fibroblast-like synoviocyte activation^87^ and provided a strong assay window.

After 30 minutes incubation with compounds, cells were treated with recombinant human TNF$\alpha$ (R&D systems) or Poly(I:C) (InvivoGen) at single EC90 concentrations, and incubated at 37$^{\circ }$C for 24 hours. TNF$\alpha$ and Poly(I:C) were chosen as they were shown to mimic innate immune activation and activation during viral exacerbations, respectively, states observed in RA and used by Jones et al.^18^. Each stimulus has been shown to induce secretion of a consistent set of six cytokines: regulated on activation, normal T-cell expressed and secreted (RANTES), Interleukin-6 (IL-6), Interleukin-8 (IL-8), Interferon $\gamma$-induced protein 10 (IP-10), Monocyte chemotactic protein 1 (MCP-1) and Growth regulated oncogene $\alpha$ (GRO$\alpha$), which recruit immune cells to the site of the disease^18^.

The following assay controls were included on each plate (quadruplicate wells): TNF$\alpha$ or Poly(I:C) alone (at selected EC90 concentration), medium alone (untreated control), positive inhibitory control (TNF$\alpha$ or Poly(I:C) + SB202190), and SB202190 alone. FLSs from three donors were tested, and FLSs from one donor were chosen since each donor displayed a robust and comparable cytokine response to each stimulus. Representative images of the cells were captured (top concentration only) to estimate compound-induced cytotoxicity based on a visual assessment of cell morphology. Supernatants were then collected from the wells and stored at -20$^{\circ }$C. Immunoassay analysis was performed using the MSD U-PLEX®format (Meso Scale Discovery, Rockville, Maryland, USA) for 5 cytokines (IL-6, IL-8, IP-10, MCP-1 and GRO) and singleplex format for RANTES. The raw MSD data were analysed in GraphPad Prism®and then the interpolated data transferred to Microsoft Excel for calculation of percentage of control values. Compounds were marked as toxic at the 10$\mu$M concentration, resulting in 30 final gene-compound pairs. Of these, drug targets initial "hits" were identified as those drug targets that produced a >50% inhibition of at least two cytokine endpoints by corresponding compounds at any concentration (10$\mu$M, 1$\mu$M or 0.1$\mu$M) under either TNF$\alpha$ or Poly(I:C) stimulation. Final "hits" were identified as drug targets that produced a >50% inhibition of at least two cytokine endpoints by corresponding compounds under both TNF$\alpha$ or Poly(I:C) by any corresponding compound at 1$\mu$M and 0.1$\mu$M, to avoid hits due to off-target effects at the highest dose (10$\mu$M).

### Evaluation metrics and statistical tests

Model performance for the analyses presented here was measured using two metrics, mean average precision (mAP) and average recall at rank *k* (Recall@k). Mean average precision is defined by:

4 $$\begin{aligned} mAP = \frac{\sum _{q=1}^{D} AP(d)}{D} \end{aligned}$$

where D is the number of diseases in the benchmark and *AP*(*d*) is the average precision for that disease, d. Average precision is defined as:

5 $$\begin{aligned} AP = \frac{\sum _{k=1}^{N}P(k)\cdot rel(k)}{{GTP}} \end{aligned}$$

where GTP is the number of ground-truth positive GeneProteins, and *rel*(*k*) is an indicator function that takes the value 1 if k is a positive example and 0 if not. *P*(*k*) is the precision at rank *k*.

Recall at is defined as:

6 $$\begin{aligned} avg. Recall@k = \frac{\sum _{d=1}^{D} Recall@k(d)}{D} \end{aligned}$$

where k is the maximum rank of interest. Average recall at rank *k* is calculated by taking the simple mean over the total number of diseases of interest, D.

The statistical test used to compare distributions across the analyses presented here was a Mann-Whitney-Wilcoxon (MWW). Medians of both distributions are reported, along with the corresponding U-statistic and p-value.

## Supplementary information

Supplementary material 1.
