# Supplementary material for: Preclinical validation of therapeutic targets predicted by tensor factorization on heterogeneous graphs
Source: Sci Rep. 2020 Oct 26;10:18250. doi: 10.1038/s41598-020-74922-z (PMC7589557; doi:10.1038/s41598-020-74922-z)
Supplement: Supplementary file 1 — Supplementary material 1. [file 41598_2020_74922_MOESM1_ESM.pdf]

Preclinical validation of therapeutic targets predicted by tensor factorization on heterogeneous graphs: Supplementary Information

Saee Paliwal*[1], Alex de Giorgio[2], Daniel Neil[1], Jean-Baptiste Michel[1], and Alix MB Lacoste[1]

[1]BenevolentAI, 1 Dock72 Way, 7th Floor, Brooklyn, NY, 11205
[2]BenevolentAI, 4-6 Maple Street, Bloomsbury, London, W1T5HD
*saee.paliwal@benevolent.ai

Knowledge Graph Edge description

Summary of graph relationships shown below:

	**Gene**	**Disease**	**GOProcess**
**Gene**	Protein-Protein Interaction (PPI) from Biogrid, SigNOR, KEGG and Reactome		
**Disease**	1. Biological Association from the GWAS CATALOG, ChEMBL, DisGeNET and from EAT. 2. Therapeutic Relationship (benchmark) from CTD, KEGG, OMIM, and LTE		
**Pathway**	Biological Association from KEGG and Reactome	Mechanistic Connection inferred from gene sets extracted from KEGG and Reactome combined with EAT Disease-Gene edges	
**Compound**	Experimental Evidence from ChEMBL (binding relations with PChEMBL value >= 7)	Therapeutic Link from Integrity (compounds tested in preclinical phases or above)	
**GOProcess**	1. Biological Association constructed from unstructured data, SVOs, and expert annotations. 2. Therapeutic Link constructed from expert annotations	Mechanistic Connection association extracted from gene set enrichment analyses from eDGAR and CTD.	Biological Association extracted from GO (is a, regulates, part of, negatively regulates, positively regulates)

Table 1. Summary of graph edges

Relationship type	EdgeCount
GO process_GO process Biological Association	143,490
Gene protein-disease Biological Association	443,330
Gene protein-GO process Biological Association	255,265
Disease-compound Therapeutic Link	13,919
Disease-pathway Mechanistic Connection	348,001
Disease-GO process Biological Association	76,587
Gene protein-pathway Biological Association	133,872
Gene protein-gene protein Interaction (PPI)	629,357
Gene protein-disease Therapeutic Relationship	128,018
Gene protein-GO process Therapeutic Link	129,382
Gene protein-compound Experimental Evidence	331,852

Table 2. Summary of Edge count by Relationship type

Entity type	Count
Compound	261,812
Disease	9,972
GOProcess	29,699
GeneProtein	18,582
Pathway	2,526

Table 3. Knowlege Graph Entity Count

Test datasets for various analyses

Below are the details of the relations, number of test edges and number of test diseases used for the analyses presented here.

Analysis	Fig/Subplot	Data splits	Test relation	Test Edge#	Test Disease#
Full Rosalind		N/A	Disease-Gene Therapeutic Relationship	24755	3722
State-of-the-art: Decoders and Dropout	Fig 3 A&B	Train-60% Valid-20% Test-20%	Disease-Gene Therapeutic Relationship	4613	198
State-of-the-art: Comparison	Fig 3 C&D	Train-60% Valid-20% Test-20%	Disease-Gene Therapeutic Relationship	1390	198
Time-slicing: Time-band	Fig 4C & 4D	Train-60% Test-40%	Disease-Gene Therapeutic Relationship		
1990				13183	1830
1995				26638	2617
2000				28407	3116
2005				20079	3082
2010				11597	2776
2015				2974	1261
Time-slicing: Forward Prediction	4E	Train-60% Valid-20% Test-20%	Disease-Gene Therapeutic Relationship	3058	184
Clinical Outcome Prediction: Failure	5A/5D	Train-60% Valid-20% Test-20%	Clinical Trial Failure	265	155
Clinical Outcome Prediction: Success	5B/5E	Train-60% Valid-20% Test-20%	Clinical Trial Success	542	338

Table 4. Details of test datasets for various analyses

Rosalind comparison with Jones et al. results

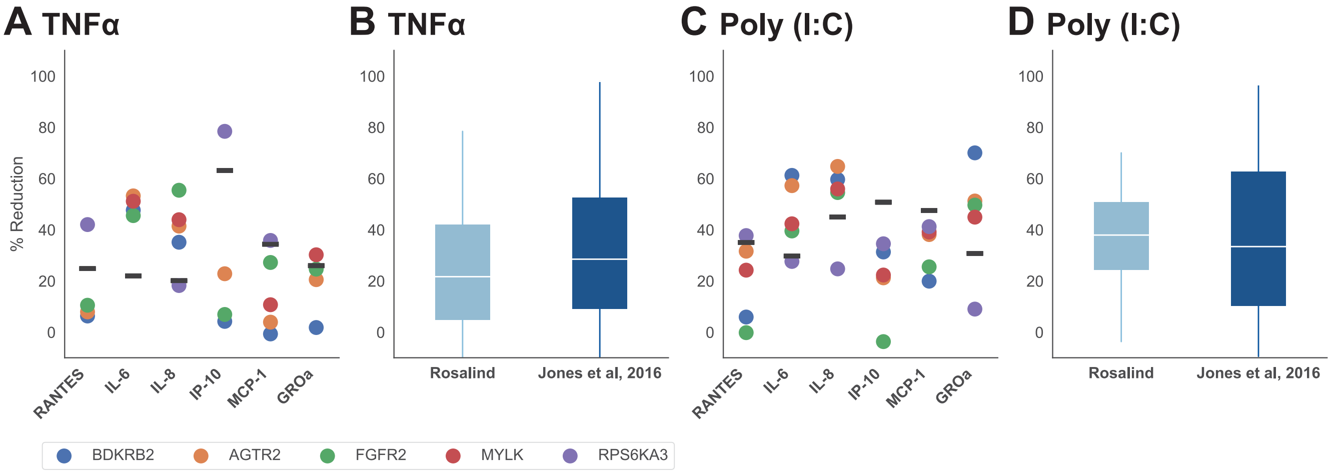

Figure 1. Performance of Rosalind Assay hits compared to the efficacy of targets in Jones et al. **A** Percent reduction by cytokine across our assay hits (colored circles) under TNFα stimulation. Black bars indicate the average percent reduction across the four target-compound pairs provided in the Supplementary Information of Jones et al.: JNKi-JNK-IN-8, p38i-PH797804, IKKi-IKK16, JAKi-tofacitinib. **B** Distribution of efficacy across cytokines for Rosalind hits versus Jones et al. targets. Plots **C**-**D** show the same comparison for Poly(I:C) stimulation.

State-of-the-art Algorithm Comparison, Additional Metrics

Reported below are the mean average precision at rank 500 (mAP@500) and recall at rank 200 (recall@200) performance numbers. Note that in the state-of-the-art comparison, we focus only on recall. We have included mAP here to provide additional information about Rosalind's relative performance, but, as we have mentioned in the manuscript, we do not believe mAP to be a reliable performance metric for these analyses.

Algorithm	mAP@500	Recall@200 Full	Recall@200 RA
**Rosalind***	**5.19**	**61.52**	**57.12**
Open Targets	2.79	42.96	41.67
SCUBA	0.72	21.66	18.42
MACAU	2.89	21.87	26.38
CATAPULT	1.32	14.56	19.30
PGCN	1.21	10.55	10.01

Table 5. State-of-the-art comparison. mAP@500 and recall@200 is calculated across the full set of 198 diseases, and reported as a value between 0 and 100. Recall@200 is also compared across all algorithms for the full set of diseases (Full) and for RA alone (RA). Recall numbers correspond to the markers shown in Figure 3C and 3D.

Aligning State-of-the-art Gene prioritization with Rosalind Data

To map diseases and gene predictions from Open Targets[1], the v3 API was used to match the disease name in the 198 disease test set to the closest match in the Open Targets database, collecting an Orphanet ID for each disease. Next, all associated genes and scores sorted according to the Open Targets composite score were collected for each disease using the API, producing a ranked list of genes for each disease in the test set. Of the 198 test diseases, 184 diseases were mapped successfully for Open Targets.

For SCUBA[2], the training genes for each of the 198 disease were provided as the seed genes for the algorithm. The algorithm learns a weighting on a matrix of gene-gene similarities, and this multiple kernel learning strategy is used to associate seed (training) genes to new genes. Five matrices were used here, as provided in their work: a Markov Diffusion Kernel inspired by heat diffusion with iteration parameters 2 and 6; and a regularized Laplacian Kernel (RLK) similar to random walks with scaling factors 1, 10, and 100. Therapeutic genes in the Rosalind training dataset were mapped to ENSEMBL[3] IDs, resulting in an 8% loss of genes which could not be mapped successfully, and used as seed genes for learning kernel weightings. After learning, these weightings were used to rank the genome. The diversity of information sources and access to the training data used in Rosalind aids the SCUBA algorithm to successfully rank genes. Of the 198 test diseases, 187 were mapped successfully for SCUBA.

For the Bayesian matrix factorization algorithm MACAU[4], the conditioning information was used from that work, using Interpro[5], Gene Ontology[6], and Uniprot[7] additional context for the genes; similarly, for diseases, literature-based disease features derived from textual term-frequency inverse-document frequency (TF-IDF) occurrences in PubMed were used in[8]. The provided textual terms were not used for the gene targets as the article material does

not provide the means to successfully map them. The disease-gene matrix was defined using the training data from the benchmark described above (using training data from Rosalind), with 10x as many randomly-sampled negative associations (zero-entries) in the matrix for every one positive entry (1-entry). This negative sampling matches the 10:1 negative-to-positive ratio used in negative sampling for ComplEx to ensure consistent positive / negative label balance across the algorithms. Of the 198 test diseases, 160 map successfully for MACAU.

Catapult[9], which relies on supervised SVMs combined with a random walk on the network, the published trained model is used to generate a matrix of 3210 diseases by 12331 genes. The OMIM IDs are mapped to internal Rosalind identifiers, and 172 of the 198 test set diseases appear in the prediction matrix.

For PGCN[10], using a graph convolutional network trained on OMIM[11] the full set of predictions were generated from the authors' shared data. This prediction matrix is 3215 diseases by 12331 genes; 66 of the 198 diseases in the test set appear in the 3215 diseases, mapping from OMIM IDs to Rosalind internal disease identifiers; 11,976 genes of the 12331 are mapped successfully. Although this algorithm has high performance for small k (approximately below 20 targets), as the authors show in their work, it suffers in ranking as k is increased and more targets are examined.

The performance across algorithms for the minimal set of diseases present in all methodologies can be found in Fig. 2, with the diseases themselves listed in Table 6.

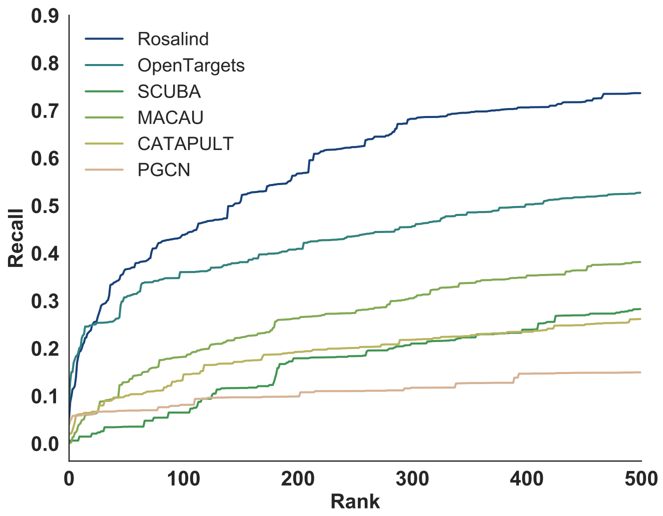

Figure 2. Performance across the minimal set of diseases present for all algorithms. All algorithms are capable of producing predictions for the 40 diseases listed in Table 6, and shown here with recall at k averaged across diseases. Note that this qualitatively matches Figure 3C.

Disease Name
Alcoholism
Alzheimer Disease
Angelman Syndrome
Anodontia
Arthritis, Rheumatoid
Attention Deficit Disorder with Hyperactivity
Autoimmune Lymphoproliferative Syndrome
Beckwith-Wiedemann Syndrome
Colorectal Neoplasms
Dyskeratosis Congenita
Ehlers-Danlos Syndrome
Esophageal Neoplasms
Gastrointestinal Stromal Tumors
Hemochromatosis
Hirschsprung Disease
Homocystinuria
Keratoderma, Palmoplantar
Leigh Disease
Leukoencephalopathies
Medulloblastoma
Migraine Disorders
Multiple Sclerosis
Nephrotic Syndrome
Obesity
Obsessive-Compulsive Disorder
Osteogenesis Imperfecta
Osteopetrosis
Pancreatic Neoplasms
Pheochromocytoma
Primary Myelofibrosis
Pseudoxanthoma Elasticum
Sarcoidosis
Severe Combined Immunodeficiency
Stomach Neoplasms
Stroke
Tetralogy of Fallot
Turcot syndrome
Urinary Bladder Neoplasms
Wilms Tumor
Zellweger Syndrome

Table 6. Minimal set of 40 diseases present for all comparison models.

References

1. Carvalho-Silva, D. *et al.* Open targets platform: new developments and updates two years on. *Nucleic acids research* **47**, D1056–D1065 (2018).

2. Zampieri, G. *et al.* Scuba: scalable kernel-based gene prioritization. *BMC bioinformatics* **19**, 23 (2018).

3. Zerbino, D. R. *et al.* Ensembl 2018. *Nucleic acids research* **46**, D754–D761 (2017).

4. Zakeri, P., Simm, J., Arany, A., ElShal, S. & Moreau, Y. Gene prioritization using Bayesian matrix factorization with genomic and phenotypic side information. *Bioinformatics* **34**, i447–i456 (2018).

5. Mitchell, A. *et al.* The interpro protein families database: the classification resource after 15 years. *Nucleic acids research* **43**, D213–D221 (2014).

6. Gene, O. *et al.* Gene ontology consortium: going forward. *Nucleic Acids Res* **43**, D1049–56 (2015).

7. Quintaje, S. B. & Orchard, S. The annotation of both human and mouse kinomes in uniprotkb/swiss-prot: one small step in manual annotation, one giant leap for full comprehension of genomes. *Mol. & Cell. Proteomics* **7**, 1409–1419 (2008).

8. ElShal, S. *et al.* Beegle: from literature mining to disease-gene discovery. *Nucleic acids research* **44**, e18–e18 (2015).

9. Singh-Blom, U. M. *et al.* Prediction and validation of gene-disease associations using methods inspired by social network analyses. *PloS one* **8**, e58977 (2013).

10. Li, Y., Kuwahara, H., Yang, P., Song, L. & Gao, X. Pgcn: Disease gene prioritization by disease and gene embedding through graph convolutional neural networks. *bioRxiv* 532226 (2019).

11. Hamosh, A., Scott, A. F., Amberger, J. S., Bocchini, C. A. & McKusick, V. A. Online mendelian inheritance in man (omim), a knowledgebase of human genes and genetic disorders. *Nucleic acids research* **33**, D514–D517 (2005).
